# Penetrating Brain Injury after Suicide Attempt with Speargun: Case Study and Review of Literature

**DOI:** 10.3389/fneur.2014.00113

**Published:** 2014-07-07

**Authors:** John R. Williams, Daniel M. Aghion, Curtis E. Doberstein, G. Rees Cosgrove, Wael F. Asaad

**Affiliations:** ^1^Warren Alpert School of Medicine, Brown University, Providence, RI, USA; ^2^Department of Neurosurgery, Rhode Island Hospital, Providence, RI, USA; ^3^Department of Clinical Neuroscience, Warren Alpert School of Medicine, Brown University, Providence, RI, USA; ^4^Brown Institute for Brain Sciences, Providence, RI, USA

**Keywords:** penetrating brain injury, ballistic injury, neurovascular injury, traumatic brain injury, intracranial antibiotic prophylaxis, seizure prophylaxis, operative timing, foreign body removal

## Abstract

Penetrating cranial injury by mechanisms other than gunshots are exceedingly rare, and so strategies and guidelines for the management of PBI are largely informed by data from higher-velocity penetrating injuries. Here, we present a case of penetrating brain injury by the low-velocity mechanism of a harpoon from an underwater fishing speargun in an attempted suicide by a 56-year-old Caucasian male. The case raised a number of interesting points in management of low-velocity penetrating brain injury (LVPBI), including benefit in delaying foreign body removal to allow for tamponade; the importance of history-taking in establishing the social/legal significance of the events surrounding the injury; the use of cerebral angiogram in all cases of PBI; advantages of using dual-energy CT to reduce artifact when available; and antibiotic prophylaxis in the context of idiosyncratic histories of usage of penetrating objects before coming in contact with the intracranial environment. We present here the management of the case in full along with an extended discussion and review of existing literature regarding key points in management of LVPBI vs. higher-velocity forms of intracranial injury.

## Introduction

Violent crime is a common occurrence in the US (1), and suicide is on the rise (2, 3); many of these involve mechanisms leading to penetrating craniocerebral injury. Wide availability of firearms and hunting weapons also allow for increased rates of accidental penetrating injury. The vast majority of these incidents in the civilian population are a result of injury due to what are considered medium-velocity missiles from low-power firearms such as handguns or hunting rifles (4). The contemporary military theater adds a large number of injuries resulting from high-velocity missiles from higher-powered rifles to the list of penetrating brain injuries affecting Americans (5). Penetrating cranial injury by mechanisms other than gunshots, however, is exceedingly rare. Congruently, literature addressing comprehensive management of low-velocity penetrating brain injury (LVPBI) is virtually non-existent.

As in the case presented here, many of the penetrating missiles and objects in LVPBI are idiosyncratic in their dimensions, as well as their method and velocity of delivery, increasing the challenge of deriving management strategies from literature on gunshot and stab PBI. Arrow-launching weapons involve projectiles traveling at velocities similar to hand-held objects in stabbings, yet they move considerably slower than missiles from firearms, resulting in intrinsic differences in the mechanics of injury (6, 7). Newer projectiles used in conventional bow-hunting as well as by underwater fishing guns are also equipped with spring-loaded barbs which eject upon contact with tissue to inflict more damage and prevent living targets from freeing themselves from an embedded arrow or harpoon. These qualities suggest this type of injury should be managed with a unique set of considerations, differing from those traditionally employed in the management of gunshot and stab wounds. No comprehensive review of penetrating craniocerebral injury by such a mechanism exists in the literature to date.

In this report, we present an unusual case of LVPBI in which a pneumatic speargun intended for fishing caused craniocerebral injury in an attempted suicide. A team of ENT surgeons worked in conjunction with a team of neurosurgeons to remove the spear in a two-part craniotomy, allowing for proximal control of the common carotid and for an anterograde extraction of the spear to prevent retrograde traction by spring-barbs. The bizarre circumstances of the injury and lack of available history surrounding the event lead to a series of unique considerations in management. Here, we also briefly review existing literature on LVPBI, comparing the mechanics of injury to higher-velocity PBI and how those mechanisms affect vascular, parenchymal, functional, and infectious sequelae. We discuss potentially critical aspects of management of such injury, including timing of foreign body removal, imaging, and antibiotic and anti-convulsant prophylaxis.

## Case Report

### History and physical examination

A 55-year-old Caucasian man with a history of depression and recently attempted suicide attempted suicide again using a fishing harpoon gun. The patient called Emergency Medical Services, but did not speak at approximately 10:00, presumably shortly after the event occurred. EMS reported to his home where they found him unresponsive with a three-foot fishing spear through his lower jaw/throat area and head, noting it to be an apparently self-inflicted injury. He was transported to a lower-level trauma center, where he was orally intubated after rapid evaluation. He was then transferred to our level-one trauma center where his surgical planning and procedure took place.

**Figure 1:**
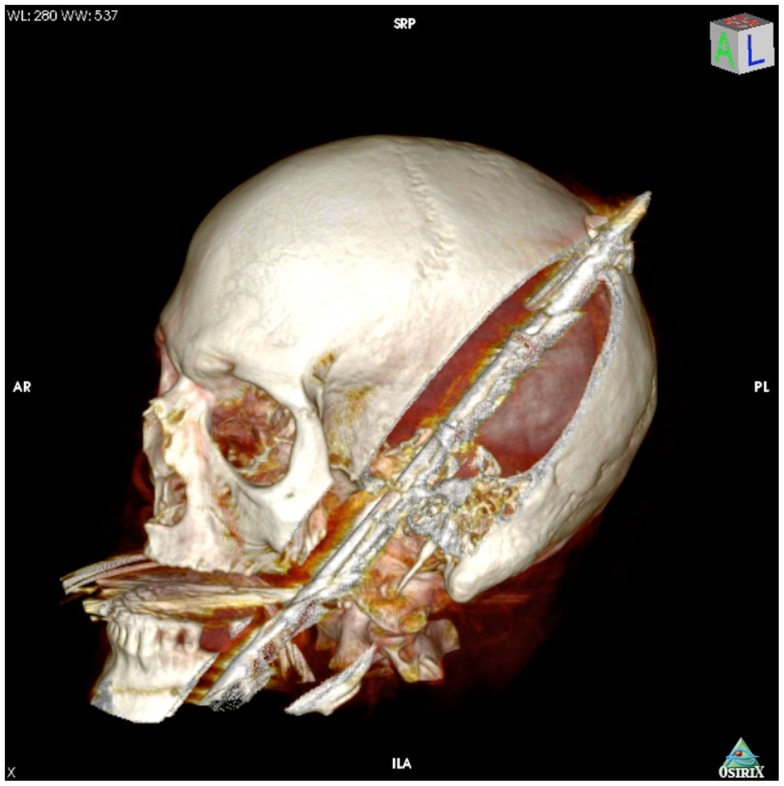
**3-dimensional computed tomography reconstruction of the spear’s tract through the oropharynx and skull**.

**Figure 2:**
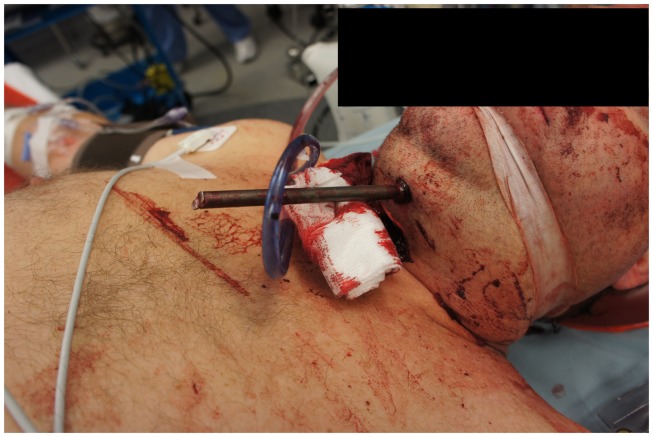
**View of the cut tail of the spear protruding from the patient’s neck before removal**.

**Figure 3:**
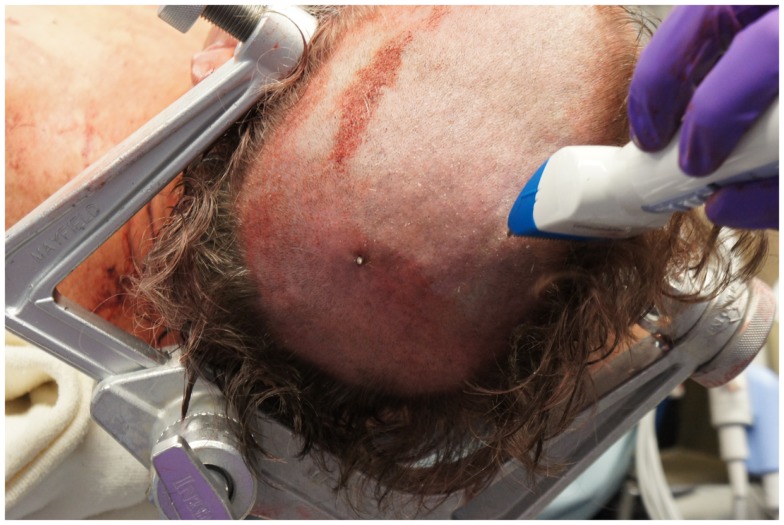
**View of the spear tip protruding through the skin overlying the left parietal bone**.

**Figure 4:**
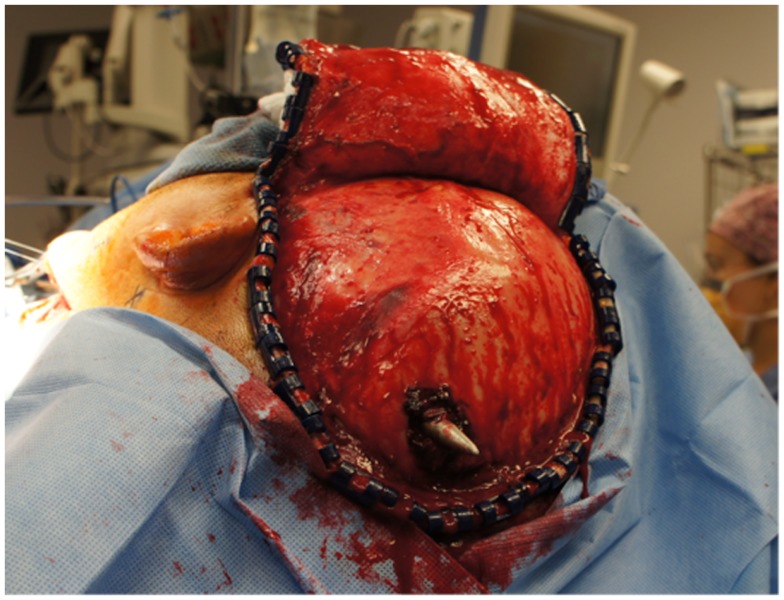
**View of the large craniotomy through a left-sided question-mark-shaped trauma flap exposing the foreign object in the posterior parietal region and allowing for a pterional-type approach to the ICA within the intracranial cavity**.

On presentation, at approximately 12:15, the patient’s Glasgow Coma Score was 3 T on chemical sedation. Along with initial primary survey, osmotherapy was administered in the emergency department (ED). Computerized tomography (CT) scan of the brain, cervical spine, and face were obtained after cutting the foreign body to allow entry into the CT gantry (Figure 4). Imaging was severely impaired by metallic artifact, as no dual-energy CT (DECT) scanner was available. Using the images obtained, the course of the foreign object was determined to proceed through the left submandibular region, oral cavity, retropharyngeal soft tissues, left petrous apex, left carotid canal, left temporal horn of the left lateral ventricle, posterior aspect of the left frontal lobe, left temporal lobe, and its partial exit through and out of the left temporoparietal region (Figures 1–4). Evidence of bone and possibly metallic fragments within the left temporal lobe along the harpoon’s tract was also observed. Intraventricular hemorrhage of the left lateral ventricle was present. CT angiography showed non-opacification of the cervical and petrous portions of the left internal carotid artery (ICA), likely related to the projectile’s path directly transecting the left carotid canal, but adequate distal filling due to collateral supply was also visualized. A retrograde thrombus of the cervical segment of the left ICA was present, as well as presumed smaller vessel injury along the projectile’s tract in the left temporal–parietal lobes and the posterior aspect of the left frontal lobe.

### Operative course

The patient was transported emergently to the operating room by the neurosurgical and otolaryngeal (ENT) surgery teams for exploration of the wounds and removal of the foreign body. Based on initial laboratory values, he was stabilized with three units of fresh frozen plasma and packed red blood cells. The patient was placed supine on the operating table, and his head was placed in Mayfield pins with head turned rightward 90°. At 14:48, the ENT team began exploring the wound in the area of the left sub-mental region and oropharynx and provided exposure to the tail end of the harpoon, as well as neck dissection for proximal control of the carotid artery. Carotid sheath contents were identified and preserved, and vessel loops were placed around the common carotid artery.

**Figure 5:**
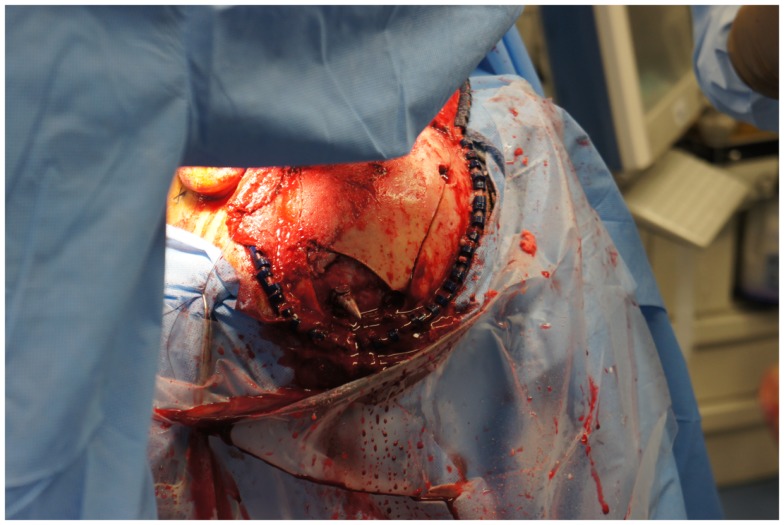
**View of the second, smaller craniotomy was formed directly surrounding the tip of the harpoon, allowing the bone to be removed by sliding it upward and posteriorly along the path of the projectile**.

The neurosurgical approach began at approximately 16:56, and included a large craniotomy through a left-sided question-mark-shaped trauma flap both to expose the foreign object in the posterior parietal region and to allow for a pterional-type approach to the ICA within the intracranial cavity; intracranial ICA exposure was required in addition to common carotid artery exposure in the neck to gain distal and proximal control of the carotid artery to prevent potential anterograde and retrograde bleeding from the site of injury in the petrous skull base. Upon exposing the skull, the harpoon tip was observed to exit at an acute angle relative to the bone, pointing posteriorly and forming a sort of latch that prevented the bone from being hinged forward to free it from the sphenoid wing. Therefore, the craniotomy was performed in two pieces: a large piece of bone was removed anteriorly providing pterional access and a second, smaller craniotomy was formed directly surrounding the tip of the harpoon, allowing the bone to be removed by sliding it upward and posteriorly along the path of the projectile (Figure 5).

**Figure 6:**
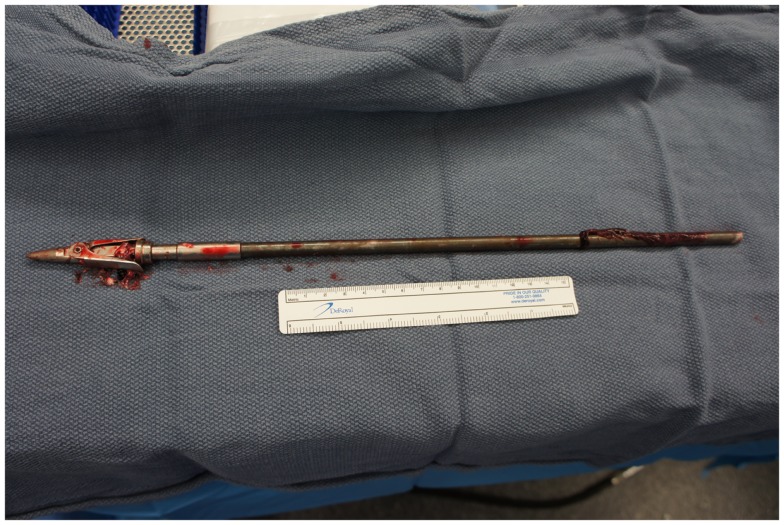
**The embedded portion of the spear after removal**.

**Figure 7:**
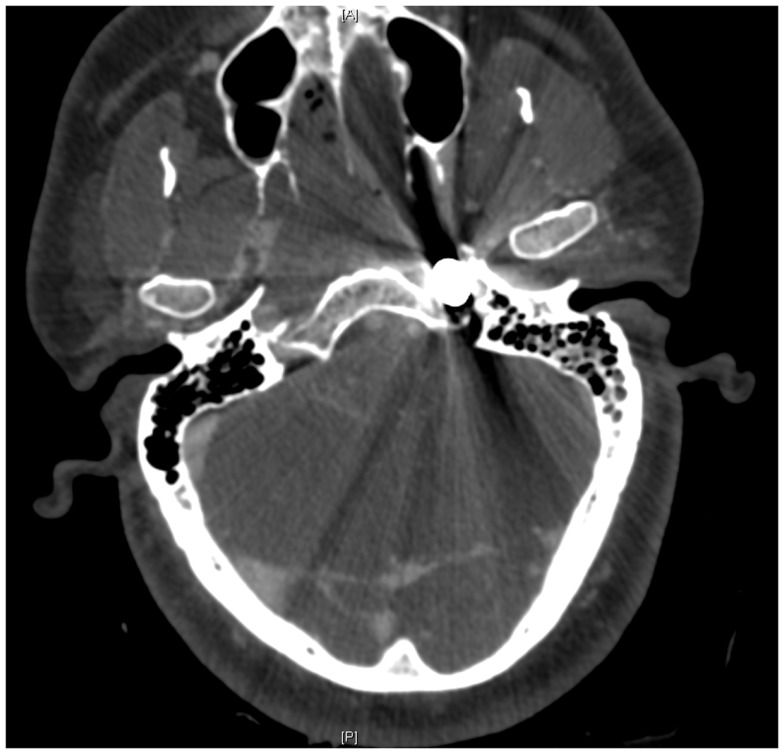
**Pre-operative CT scan showing complete transection of the petrous portion of the left internal carotid artery**.

**Figure 8:**
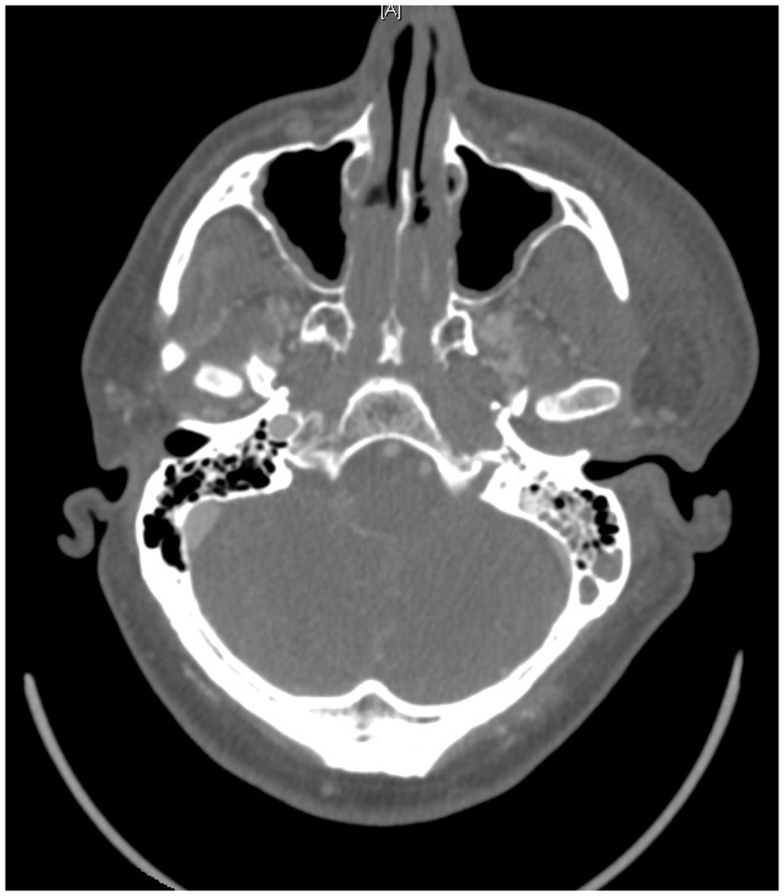
**Post-operative follow up CT scan showing lack of flow through the left ICA**.

Upon removing the skull, the dura was noted to be surprisingly lax, suggesting minimal secondary cerebral edema. The distal ICA was exposed by splitting the Sylvian fissure and following the olfactory nerve to the optic nerve, leading to the artery. Clearance for the placement of an aneurysm clip was created and a clip was selected and placement rehearsed, but not actually performed at this point. Once the ENT surgical team signaled proximal exposure and preparation of the ICA for proximal control was complete, the harpoon was gently extracted anterograde along the original trajectory in order to avoid damage from its barb-like fins with a grain against the retrograde direction at approximately 17:30, 7.5 h after the initial event (Figure 6). When it was fully removed, the patient was observed for bleeding, and, remarkably, there was none noted from the cortical exit site, the entry site in the neck, or the oropharynx (Figures 7 and 8). Hemostasis was obtained along craniectomy margins, whereupon both bone pieces were reinserted and plated to the skull with titanium fixtures.

### Post-operative course

The patient was initially placed on a triple antibiotic regimen of ceftriaxone, vancomycin, and metronidazole for prophylaxis against microorganisms carried by a harpoon with an unknown history of use that also traversed the oral cavity before entering the cranium. The infectious disease team deemed it best to wait for signs of necrotizing infection before broadening coverage to include oral flora and organisms associated with freshwater exposure (*Aeromonas, Edwardsiella tarda*) and potential salt-water organisms (*Vibrio vulnificus*).

In the days following the removal of the harpoon, the patient remained intubated in the ICU, demonstrating a consistently improving neurological exam. He was able intermittently to follow commands, and to move the left side of the body, though the right side remained hemiplegic. Despite broad-spectrum antibiotics, the patient developed a persistent low-grade fever without a clear extracranial source, suggesting a possible intracranial infection. An MRI was obtained demonstrating multiple areas of diffusion restriction in the temporal lobe as well as ring enhancement and diffusion restriction adjacent to the harpoon tract, raising suspicion for an abscess along the tract.

Eleven days after the initial surgery to remove the harpoon, the patient returned to the operating room for wound exploration and potential abscess drainage. Intra-operatively, no evidence of empyema was observed and no purulent fluid could be aspirated from within the harpoon tract, however, the brain parenchyma surrounding the exit site did appear necrotic, suggestive of cerebritis. The site of entry into the medial temporal lobe was explored more directly, and a large cavity was found in the left middle temporal gyrus. This space was entered and non-purulent fluid and CSF were aspirated without evidence of a purulent collection. The area was thoroughly irrigated and an external ventricular drain (EVD) was placed in the superior tract. A small quantity of clear fluid and a dark clot with a slightly murky appearance was aspirated, possibly reflecting a low-grade infection. The EVD was left in place, the bone flap replaced and the incision closed, and the patient returned to the ICU.

Because no organisms were isolated, perhaps due to the ongoing antibiotic therapy, broad-spectrum treatment was continued with meropenem and vancomycin. His neurological exam continued to improve and he was successfully extubated. He was observed to have spontaneous movement in his left lower and upper extremities, and began to recover some strength in his lower right extremity. He was able to follow simple commands, and was attempting to verbalize. However, on hospital day 23, based on the family’s interpretation of the patient’s wishes, and after approval from the hospital ethics committee, the patient’s code status was made DNR/DNI and eventually transitioned to comfort measures only. He remained stable over the coming days with resolving fevers and oxygenating well on room air. Eventually, he was discharged to hospice care at the request of his family, where without tube-feeds and other medical support, he passed away 33 days after the initial suicide attempt.

## Discussion

We present here an interesting case of penetrating cranial injury by a fishing harpoon. Given the rarity of this type of injury, there are no specific guidelines for their management. There have been a number of attempts to provide comprehensive guidelines for the management of penetrating brain injury of all kinds (8–10). However, these guidelines have been largely informed by data from gunshot wounds, which account for the vast majority of PBI in the US. Here, we discuss differences between low- and high-velocity PBI that could warrant deviations from established standards of neurosurgical and intensive care.

### Tissue damage and the ballistics of the projectile

Injury from low-velocity projectiles differs from that of medium- and high-velocity projectiles not only quantitatively but also qualitatively. From the kinetic energy formula (*K* = 1/2 mv^2^), the amount of tissue damage depends on the amount of energy transferred to that tissue, making high-velocity missiles from guns potentially much more devastating than more massive objects held in hands (11, 12). High-velocity projectiles are defined as traveling greater than 2,000 ft (609.6 m) per second, whereas information on the exact cutoff between medium-velocity and low-velocity is not as clearly defined (7, 12). Most spearguns, which are at the higher end of low-velocity projectiles, will list muzzle velocities under 200 ft/s (61 m/s) (4). This speargun muzzle velocity is on the order of a 100 mile/h fastball from a baseball pitching ace, which translates to 146.7 ft/s (44.7 m/s). The fastball serves a good approximation of the upper range of velocity for a hand-held object plunged into the cranial vault.

In high- and medium-velocity penetrating trauma, the increased kinetic energy delivered to tissues results in radial stretching and cavitation that cause shear forces leading to profound and widespread axonal disruption and endanger larger areas of microvasculature (6, 11). Bullet yawing – deviating from the short to long axis while traveling through tissue – adds to the damage rendered by medium- and high-velocity missiles (6). Low-velocity processes do not transfer the same order of magnitude of kinetic energy or blast effect to surrounding tissues, implying that the at-risk “real estate” is largely or entirely limited to those neurons and axonal pathways in the direct path of the projectile (6, 9, 11, 13, 14).

### Considerations in history-taking

Emergent and thorough physical exam and laboratory studies should be performed according to well-established guidelines in PBI (9, 15–17). However, LVPBI patients warrant extra care in history-taking as the mechanism of injury frequently reflects objectionable and/or illegal behavior. The case presented here and another with an injury by the same mechanism are examples of such behavior in the form of failed suicide attempts (18). Other cases of LVPBI have involved unsupervised children playing with harpoon guns similar to the one used by the patient described in this report (19, 20), a young man constructing a homemade explosive device (14), and poorly attended children falling with pointed objects in hand (15, 21). All of these circumstances are of potential concern to law enforcement and social welfare organizations, making accurate documentation of the circumstances surrounding the history of present illness even more important.

Furthermore, all witnesses and individuals who were around the patient near the time of injury should also be questioned, as the injuries often impair the patients’ language and/or memory function. Early identification and treatment of injuries is paramount in preserving function in patients, yet the nature of many of these patients’ injuries inherently renders them unreliable providers of information. This is especially important in patients where the site of entry is small, subtle, and/or concealed by hair (13), and in cases of possible retained objects that are MRI contraindications or poorly visualized by CT, like large metal fragments (14) and wood (15, 21), respectively. Thorough physical examinations of the scalp and hair should be performed, noting trauma, markings, and any serous or sanguineous fluids oozing from the entry site (15). As in the case presented above, the identification of an exit site could play an important role in the surgical approach to removal, especially when retrograde-aligned barbs or wooden splinters are present.

### Imaging in LVPBI

Imaging in PBI involving gunshot wounds almost uniformly begins with CT to locate the bullet and associated retained fragments (15, 22). Pre-operative radiographic studies can be difficult to obtain and interpret in cases involving retained foreign bodies after LVPBI, as penetrating objects must have high mass and density to pierce the skull at low velocities. In cases where wooden fragments are suspected, MRI can resolve wood from similarly hypodense soft tissues (15, 21). However, most cases tend to involve large, metallic objects, such as knives, nails, screws, or harpoons, and are hyperdense relative to biological tissues. Metal generates CT artifact that obscures anatomy crucial to planning surgical approaches, and it is contraindicated in MR imaging.

When available, DECT scanning with 3-D reconstruction may be of added benefit in further understanding the trajectory of the projectile and the structures damaged by it. DECT has been shown to reduce metallic artifact, and one study by Yu et al. suggests that DECT used in adult imaging can produce diagnostic images of similar or improved quality compared to conventional single-energy scans with the same radiation exposure (23, 24). DECT offers further benefit in its ability to resolve microvascular perfusion, allowing neurosurgical teams to better evaluate areas of viable parenchyma before planning the extent of debridement in procedures following the initial removal (25, 26).

As with any PBI, cerebral angiography is recommended with suspicion of high-risk vascular injury, sub-arachnoid hemorrhage, or signs of hematoma (10, 22, 27, 28). In the case presented here, a pre-operative CT angiogram demonstrated severe carotid injury, while the post-op. catheter angiogram showed complete left petrous-ICA cutoff with total cross-filling from the right ICA. Acutely, it might be quite difficult to identify a traumatic vessel injury, especially if it is surrounded by significant clot. As was done in this case, we feel it is reasonable to repeat an angiogram at 7–10 days post-op. to ensure the patient is not developing a traumatic pseudoaneurysm or AV fistula, typically caused by scattered low-velocity bone fragments (10, 22, 29). Such lesions may not become apparent until 5–10 days post injury, or in cases with significant hematoma or tamponade, may not become evident for 14–21 days.

### Timing and strategy of foreign body removal

In general, penetrating brain injury requires prompt surgical attention, and removal of accessible foreign bodies within 12 h (10, 30). LVPBI like all other PBI requires timely removal of foreign bodies, and patients with active intracranial bleeding should be moved toward tamponade and/or decompression as quickly as possible (30, 31). However, in cases such as the one presented here where patients are hemodynamically stable with little or no active bleeding, there may be benefit in allowing for more time between admission and surgical intervention. In the presence of vascular injury, immediate removal of the projectile may initiate a fatal exsanguinating event or change pressure gradients such that watershed areas supplied by anastomotic cross-filling will infarct in the time it takes to control bleeding. For example, in our case, the seven or more hours required to transfer the patient to a level-1 trauma center, cut the harpoon, obtain appropriate imaging, transport the patient to the OR, and expose regions of interest before removing the harpoon, may have allowed sufficient time for the tamponade of the carotid artery by the harpoon, resulting in a clot stable enough to buffer the high pressures of the ICA to prevent bleeding upon removal.

Great care must be exercised in the removal of manipulation of penetrating objects with extracranial extensions such as knife handles or harpoon tails. Knives pulled from the victim of stabbings by the perpetrator or inexperienced medical team members in the field tend to be jarred and twisted unnecessarily, causing additional trauma to affected structures. Penetrating knives removed by trained physicians in a fully equipped medical care setting have been shown to have better outcomes than those pulled in the field (32, 33). A portion of the penetrating objects can remain partially outside of the cranial vault. In these cases, extra measures will be necessary to minimize further inoculation of brain tissue with toxic microorganisms via manipulation of the penetrating object or the need to push initially external projectile material through brain parenchyma in order to remove the entire missile in an anterograde fashion. In such instances, extreme caution should be taken to cut or reduce the penetrating object remaining outside the skull leaving only enough to manipulate the projectile according to a pre-determined surgical plan. In accordance with the logical principles and data that require surgical fields and instruments to be as sterile as possible, any external portions of the projectile that must pass through tissue inside the vault should be cleaned and sterilized as thoroughly as possible.

In general, LVPBI extraction techniques depend on the site of penetration and physical morphology of the penetrating object. Asymmetric geometry can pose exceptional difficulty in determining which exit path will minimize damage from removal by balancing routes that traverse the least essential parenchymal territories with the exit direction that would present the object’s dimension of smallest diameter (6, 14). In this case, surgical removal in the anterograde direction of the projectile was the safest form of treatment. The few precedents for the removal of a fishing harpoon from the cranium in the literature also advocated anterograde removal (5, 18). Improvements in the efficacy of fishing spears have hinged upon the addition of spring-loaded blades and barbs, which deploy upon contact with a higher density material: typically and hopefully fish flesh. The grain of these blades runs against as the harpoon’s trajectory, ensuring additional damage to brain parenchyma and vasculature with a retrograde approach.

### Mechanisms of injury

Vascular complications have been reported to range from 5 to 40% (27, 33) in both high- and low-velocity penetrating injury, with traumatic aneurysm formation being the most commonly reported vascular injury (10, 14, 27, 29, 32–36). True aneurysms have been reported in high-velocity PBI, but the majority of these vascular injuries take the form of pseudoaneurysms, though this is based on data from high-velocity head wounds in wartime (10, 37–39), and the incidence has not been well-documented in low-velocity PBI. Other common vascular injuries in high- and low-velocity PBI include arteriovenous fistulas, immediate and delayed, sub-arachnoid hemorrhage (12, 13, 32, 34, 36, 40, 41), and ischemic damage secondary to vasospasm (9, 10, 12, 32, 34, 36, 42–44). Earlier guidelines for the management of PBI suggested pre-operative cerebral angiography is only indicated when vascular injury is suspected (9, 10, 12, 32, 34, 36). However, more recent data from conflicts in the Middle East suggest more than one-third of all penetrating brain injury may result in traumatic intracranial aneurysm formation (45, 46). Thus, pre-operative CTA may be warranted in all cases of PBI, including low-velocity.

Cortical damage from penetrating injury leading to gliosis and glial scar also predisposes to seizure. Increased severity of injury as determined by the Glasgow outcome scale grade is associated with increased risk of seizure (9, 47). In all cases of PBI, 30–50% of patients reported seizures (10, 47). Of those cases, the vast majority developed seizures within the first 2 years after injury, but up to 18% did not have seizures until 2–5 years after sustaining penetrating injury (10, 47, 48). Studies have suggested there is benefit from using prophylactic anticonvulsants such as phenytoin, carbamazepine, valproate, or phenobarbital within the first 7 days after the event (10, 47, 49). Some debate exists over the utility of continuing prophylaxis beyond the first week, but newer evidence suggests that, given the high likelihood of developing seizure complications, the psychosocial benefits of continued use of anticonvulsants outweigh the risks of the adverse drug reactions in all but the most minimally injured patients (9, 10).

### Management of potential infection

No single standard of care has emerged surrounding PBI as infection control practice varies greatly among different departments and institutions (9, 10). Several uncertainties remain regarding timing of antibiotic use, length of antibiotic regimen, and whether the early or prophylactic use of antibiotics produces more resistant strains of bacteria. Recent pan-PBI guidelines have suggested a course of ceftriaxone, metronidazole, and vancomycin for 7–14 days (9, 10). However, some literature suggests that unless a specific colony has been identified, or specific clinical scenario (i.e., mucosal involvement) dictates, prophylactic antibiotics should not be used (9, 10, 50). There is a definitive lack of good data from randomized controlled trials, and further research is needed to determine the best course of action.

As in this case, consultation with the infectious disease service can be helpful to tailor a treatment that is specific to the type of injury, risks specific to the local ecosystem, and the patient’s comorbid risk profile. In the case presented here, prophylaxis against specific risks related to the patient’s history were withheld until evidence of necrotizing infection was present on imaging after several days of a sustained, low-grade fever. This patient had few social contacts and lived alone, preventing the medical team from gathering a complete history. Historical details on the spear’s exposure to fresh and/or salt-water as well as other relevant information regarding the patient’s diet and dentition would have been useful in tailoring a more individualized antibiotic regimen. This underscores the need for rigorous history-taking, as it is essential in guiding antibiotic prophylaxis and stewardship.

## Conclusion

In this unusual case of penetrating pharyngio-cranial injury from a fishing spear, a number of unique problems in management were posed, as low-velocity penetrating intracranial injuries are rare, and definitive guidelines are lacking in the literature. Based on our review and experience with the case presented here, we suggest: DECT scanning as the initial radiographic modality; pre-operative CT angiography study in all cases of LVPBI with immediate post-op. and follow-up angiography 7–14 days post-op.; a delayed surgical extraction that allows time for appropriate imaging and tamponade by the penetrating object; history-centered and individualized antibiotic prophylaxis; and prophylactic anticonvulsants. Data are lacking in the management of LVPBI, and prospective studies comparing delayed interventions and prophylactic drug strategies will be key in preparing definitive guidelines for management. Further insight into the true mechanisms of injury to brain tissue, superiority of craniotomy or craniectomy in initial decompression, differences in missile materials on coagulation and infectious properties, and the most useful imaging techniques and algorithms will be necessary to optimize those guidelines.

## Conflict of Interest Statement

The authors declare that the research was conducted in the absence of any commercial or financial relationships that could be construed as a potential conflict of interest.
